# Intelligent grading of sugarcane leaf disease severity by integrating physiological traits with the SSA-XGBoost algorithm

**DOI:** 10.3389/fpls.2025.1698808

**Published:** 2025-10-15

**Authors:** Xinrui Wang, Jihong Sun, Peng Tian, Mengyao Wu, Jiawei Zhao, Jiangquan Chen, Ye Qian, Canyu Wang

**Affiliations:** 1 College of Big Data, Yunnan Agricultural University, Kunming, Yunnan, China; 2 College of Information Engineering, Kunming University, Kunming, Yunnan, China; 3 Qujing Tobacco Company, Qujing, Yunnan, China

**Keywords:** sugarcane leaf diseases, disease severity grading, physiological traits, machine learning classification, hyperparameter optimization

## Abstract

**Introduction:**

Accurate assessment of sugarcane leaf disease severity is crucial for early warning and effective disease control.

**Methods:**

In this study, we propose an intelligent method for identifying sugarcane foliar disease severity based on physiological traits. Field-collected data—including Soil and Plant Analyzer Development (SPAD) values, leaf surface temperature, and nitrogen content—were acquired using a plant nutrient analyzer (TYS-4N) from sugarcane leaves infected with brown stripe disease, ring spot disease, and mosaic disease at four severity levels (mild, moderate, moderately severe, and severe). After min-max normalization, six classification models—KNN, AdaBoost, Random Forest (RF), Logistic Regression (LR), Decision Tree (DT), and XGBoost—were developed, and the Sparrow Search Algorithm (SSA) was employed to optimize hyperparameters for enhanced performance.

**Results:**

Results demonstrate that SSA significantly improved the classification capability of all models. The SSA-XGBoost model achieved the best performance, with Precision, Recall, F1 Score, and Accuracy all exceeding 0.9186, and a comprehensive PRFA score of 0.9326. When validated on an independent dataset from Gengma County, the model achieved an overall accuracy of 0.91, indicating strong generalization ability and field applicability.

**Discussion:**

Compared to image-based deep learning approaches, the proposed method offers advantages in terms of data accessibility, computational efficiency, and model transparency, making it well-suited for rapid on-site diagnosis in agricultural settings. This study provides an efficient and reliable technical framework for intelligent diagnosis and early warning of sugarcane disease severity.

## 1 Introduction

Sugarcane is one of the most important sugar crops worldwide, accounting for approximately 75% of global sugar production (Qaadan et al., 2025; Kurniawan et al., 2025). However, the frequent occurrence of foliar diseases severely threatens sugarcane growth and development, leading to yield loss, reduced sugar content, and significant economic losses (Bao et al., 2024). Therefore, accurate identification of disease severity is not only essential for timely control measures and crop health maintenance, but also critical for maximizing yield potential, optimizing sugar accumulation, and promoting sustainable agricultural development.

In recent years, the integration of artificial intelligence and sensing technologies has opened new pathways for intelligent diagnosis of crop diseases. Researchers have extensively explored deep learning models based on image analysis, achieving notable success in various crop disease recognition tasks. Early approaches often combined convolutional neural networks (CNNs) with traditional classifiers. For instance, Shradha et al. (2023) extracted features using multiple CNNs and employed a support vector machine (SVM) for classification, achieving an accuracy of 82.80% in plant disease detection. As model architectures evolved, the You Only Look Once (YOLO) series demonstrated strong performance in object detection tasks; Kalezhi and Shumba (2025) achieved over 80% mean average precision (mAP) in cassava disease detection. For sugarcane-specific diseases, Sun et al. (2023) proposed the SE-Vit hybrid network, achieving an accuracy of 89.57%, while Hong et al. (2024) improved a VGG-16-based model to achieve a high accuracy of 98.89%. To further enhance model performance, attention mechanisms and optimized loss functions have been introduced. Sun et al. (2024) integrated the Efficient Multi-Scale Attention (EMA) attention mechanism and focal loss into YOLOv8, effectively mitigating issues of complex backgrounds and sample imbalance in field images. Moreover, the application of Transformer architectures has pushed performance boundaries; Kuppusamy et al. (2024) achieved a classification accuracy of 98.5% in sugarcane leaf disease recognition using a Hybrid Shifted Vision Transformer.

Meanwhile, researchers have begun to transcend the limitations of single-modality imaging by exploring diagnostic methods that fuse multi-source information to improve the scientific rigor and robustness of assessments. Hyperspectral imaging has gained attention due to its sensitivity to plant biochemical parameters. Bao et al. (2024) combined hyperspectral data with deep neural networks to enable early detection of sugarcane smut, achieving over 90% accuracy. Pereira et al. (2025), in a systematic review, noted that 88% of related studies employed hyperspectral technology, often combined with vegetation indices (VIs) and principal component analysis (PCA), with classification accuracies generally exceeding 71%. Poblete et al. (2023) utilized high-resolution satellite data to detect vascular disease symptoms in trees, extending the application of remote sensing to large-scale monitoring. Additionally, Gianni and Maridina (2021) proposed a multi-output learning framework to simultaneously diagnose disease types and stress severity, enhancing the comprehensiveness of assessment. Adluri and Bhukya (2025) further incorporated gene expression data into predictive modeling, achieving 96.16% accuracy in rice disease early warning using their adaptively optimized residual long short-term memory with multilayer perception (AO-RLSTM-MLP) model, enabling detection of asymptomatic infections.

Despite their strong performance under controlled conditions, these technologies face multiple challenges in real-field applications. First, environmental interference significantly affects model performance: variations in illumination, leaf overlap, and background noise degrade image quality, causing the accuracy of hyperspectral models to drop from over 90% in laboratory settings to below 70% in field conditions (Abbas et al., 2023; Pereira et al., 2025). Moreover, the high cost and complex calibration requirements of hyperspectral equipment limit their adoption among smallholder farmers (Kurniawan et al., 2025). Second, most disease severity assessments still rely on lesion area or visual scoring (Qin et al., 2025), making it difficult to dynamically reflect changes in plant physiological status (e.g., chlorophyll and nitrogen levels). Although Vasavi et al. (2023) used models such as random forest and AdaBoost to predict chili diseases, and Bin et al. (2023) proposed the triple-branch Swin Transformer classification (TSTC) network to simultaneously classify disease and severity, their inputs remain limited to image features, lacking integration with physiological parameters. Furthermore, model optimization is inefficient: traditional grid search or random search incurs high computational costs (Sharma et al., 2025), and hybrid optimization algorithms (e.g., the Hybrid WOAAPSO algorithm, which merges Adaptive Particle Swarm Optimization (APSO) with the Whale Optimization Algorithm (WOA) by Vijayan and Chowdhary, 2025) still face challenges in convergence speed within high-dimensional parameter spaces. The stacked ensemble framework proposed by Qaadan et al. (2025) improved classification performance but relied on resource-intensive models, limiting its deployability in edge environments.

To address these challenges, this study proposes a novel approach for assessing sugarcane leaf disease severity using plant physiological traits and intelligent optimization algorithms. The main research contributions are:

1. Field Data Collection and Dataset Construction: To overcome the environmental adaptability issues associated with traditional image-dependent methods, this study employs a portable plant nutrient analyzer (TYS-4N) to collect non-image physiological data from sugarcane leaves in the field. By measuring SPAD values, leaf surface temperature, and nitrogen content, we constructed a comprehensive dataset covering various sugarcane diseases (brown stripe, ring spot, and mosaic) at different severity levels.
2. Machine Learning Model Optimization Using SSA: To address the inefficiency of traditional hyperparameter tuning methods, we employed the SSA to optimize six mainstream machine learning models (KNN, AdaBoost, Random Forest, Logistic Regression, Decision Tree, and XGBoost). We introduced a composite evaluation metric PRFA, consisting of Precision, Recall, F1 Score, and Accuracy, to comprehensively assess model performance. The objective of SSA optimization was to maximize the PRFA score on the validation set, thereby enhancing model robustness and generalization.
3. Physiological Trait-Based Disease Severity Assessment Model: Based on the optimized models, we developed a disease severity assessment model centered on SPAD values, leaf surface temperature, and nitrogen content. The input layer directly maps physiological features, while the output layer adopts a multi-classification strategy (mild, moderate, moderately severe, and severe) to identify disease severity. To validate model robustness, cross-regional testing was conducted in sugarcane fields in Gengma County, Yunnan Province.

This study not only provides a new approach for intelligent identification of sugarcane disease severity but also offers methodological support for digital management of diseases in other crops, demonstrating significant theoretical innovation and practical value.

## 2 Materials and methods

### 2.1 Data collection and preprocessing

**Figure 1:**
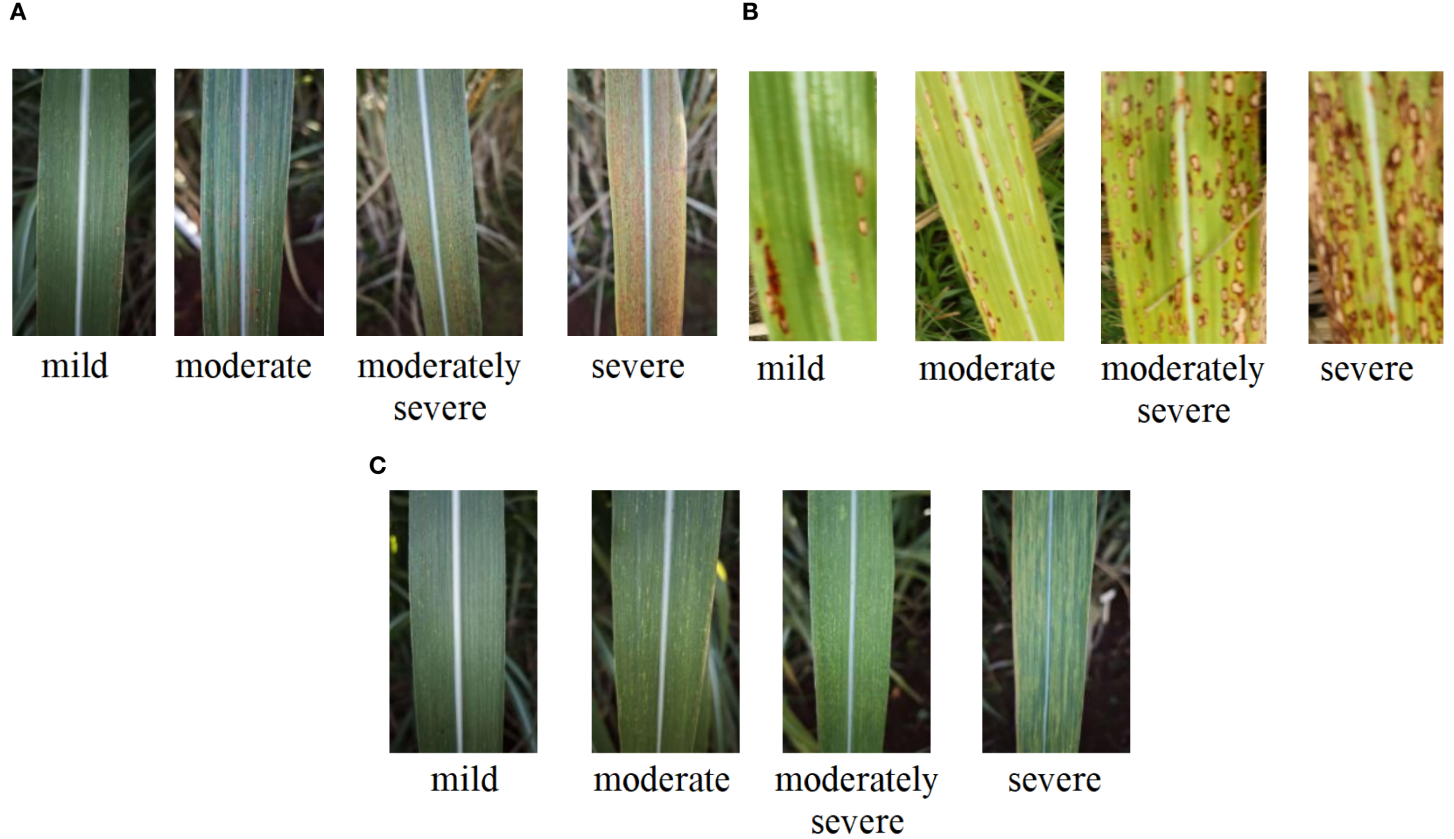
Representative symptoms of sugarcane leaf diseases at different severity levels: **(A)** Brown Stripe, **(B)** Ring Spot, and **(C)** Mosaic.

The data used in this study were collected from two representative sugarcane cultivation sites in Yunnan Province, China. The primary dataset was obtained from the sugarcane germplasm resource nursery/breeding station of Yunnan Agricultural University, where two cultivars—Dianzhe and Xintaitang—were planted. Prior to data collection, disease severity levels for brown stripe disease, ring spot disease, and mosaic disease were systematically classified based on expert consultation and field observations. The assessment was conducted by evaluating visual symptoms on green leaves, including lesion morphology (size, number, spatial distribution, and color change), and disease severity was categorized into four levels: mild, moderate, moderately severe, and severe (see **Figure 1** and **Supplementary Table 1**).

Physiological parameters were measured using a portable plant nutrient analyzer (TYS-4N, Top Cloud-Agro Technology, China). For each infected leaf, three measurements of SPAD value (indicating chlorophyll content), leaf surface temperature, and nitrogen content were taken at different locations within the lesion area. The average of the three readings was recorded as the representative value for that sample. The corresponding disease severity level was also documented for each measurement. Data were collected during the early maturity stage of sugarcane in November 2024, resulting in a total of 2,212 valid samples: 343 mild, 628 moderate, 670 moderately severe, and 571 severe cases.

To evaluate the model’s generalization capability, an independent validation dataset was collected from the Gengma Sugarcane Plantation, the largest sugarcane production base in Yunnan Province, primarily cultivating the Dianzhe variety. The same measurement protocol—identical severity grading criteria and instrument settings—was strictly followed. Data collection was completed in December 2024, yielding 635 validation samples: 28 mild, 63 moderate, 127 moderately severe, and 417 severe. The geographical, climatic, and agronomic differences between the two sites enhance the robustness of the model and enable rigorous cross-regional and cross-ecological validation.

The dataset used in this study comprises three physiological variables measured from diseased sugarcane leaf regions—SPAD value, leaf surface temperature, and nitrogen content—along with a categorical label indicating disease severity, classified into four levels: mild, moderate, moderately severe, and severe. To facilitate model training and evaluation, the severity labels were numerically encoded using ordinal encoding: “mild” was assigned 0, “moderate” → 1, “moderately severe” → 2, and “severe” → 3. An example of the preprocessed dataset is presented in **Table 1**.

**Table 1:** Example data of SPAD values, leaf surface temperature, nitrogen content, and disease severity.

Number	SPAD	Leaf surface temperature	Nitrogen	Target
1	7.6	20	2.8	3
2	46.6	21.37	14.5	1
3	32.3	21.37	10.2	3
…	…	…	…	…
2210	49.8	20.31	15.5	0
2211	44.4	20.31	13.9	1
2212	40.8	20.31	12.8	2

### 2.2 Model selection and hyperparameter optimization

To address challenges such as limited sample size and class imbalance inherent in the dataset, six representative machine learning algorithms were systematically selected and comparatively evaluated: KNN, a non-parametric method that classifies samples based on majority voting among their nearest neighbors (Parthasarathy and Chatterji, 1990); AdaBoost, an adaptive boosting algorithm that dynamically adjusts sample weights to focus on hard-to-classify instances (Schwenk and Bengio et al., 2000); RF, an ensemble of decision trees built using bagging, offering strong generalization capability; Logistic Regression (Breiman, 2001), LR, a simple and interpretable linear classifier (Singh et al., 2009); DT, a model that makes decisions based on tree-structured rules—easy to interpret but prone to overfitting (Geibel et al., 2002); and XGBoost, an efficient and regularized gradient boosting framework that delivers state-of-the-art performance across a wide range of machine learning tasks (Chen and Guestrin, 2016). These models span linear classifiers, instance-based learning, and ensemble learning frameworks, enabling a comprehensive assessment of the mapping between physiological features and disease severity across diverse hypothesis spaces, thereby ensuring robustness and representativeness in model selection.

Some of the selected models inherently possess a certain degree of robustness to class imbalance due to their algorithmic mechanisms. For instance, ensemble-based methods such as Random Forest and XGBoost mitigate class bias to some extent by constructing multiple base learners and incorporating randomness or gradient-based optimization. AdaBoost, on the other hand, dynamically adjusts the weights of misclassified samples, thereby placing greater emphasis on minority-class instances that are difficult to classify. Given the limited overall sample size, resampling techniques—such as oversampling (e.g., SMOTE) and under sampling—are prone to causing overfitting under small-sample conditions and may hinder model generalization. Furthermore, in model evaluation, we primarily rely on metrics robust to class imbalance, such as the F1-score and recall, rather than accuracy alone, to ensure the objectivity and reliability of our assessment results.

**Figure 2:**
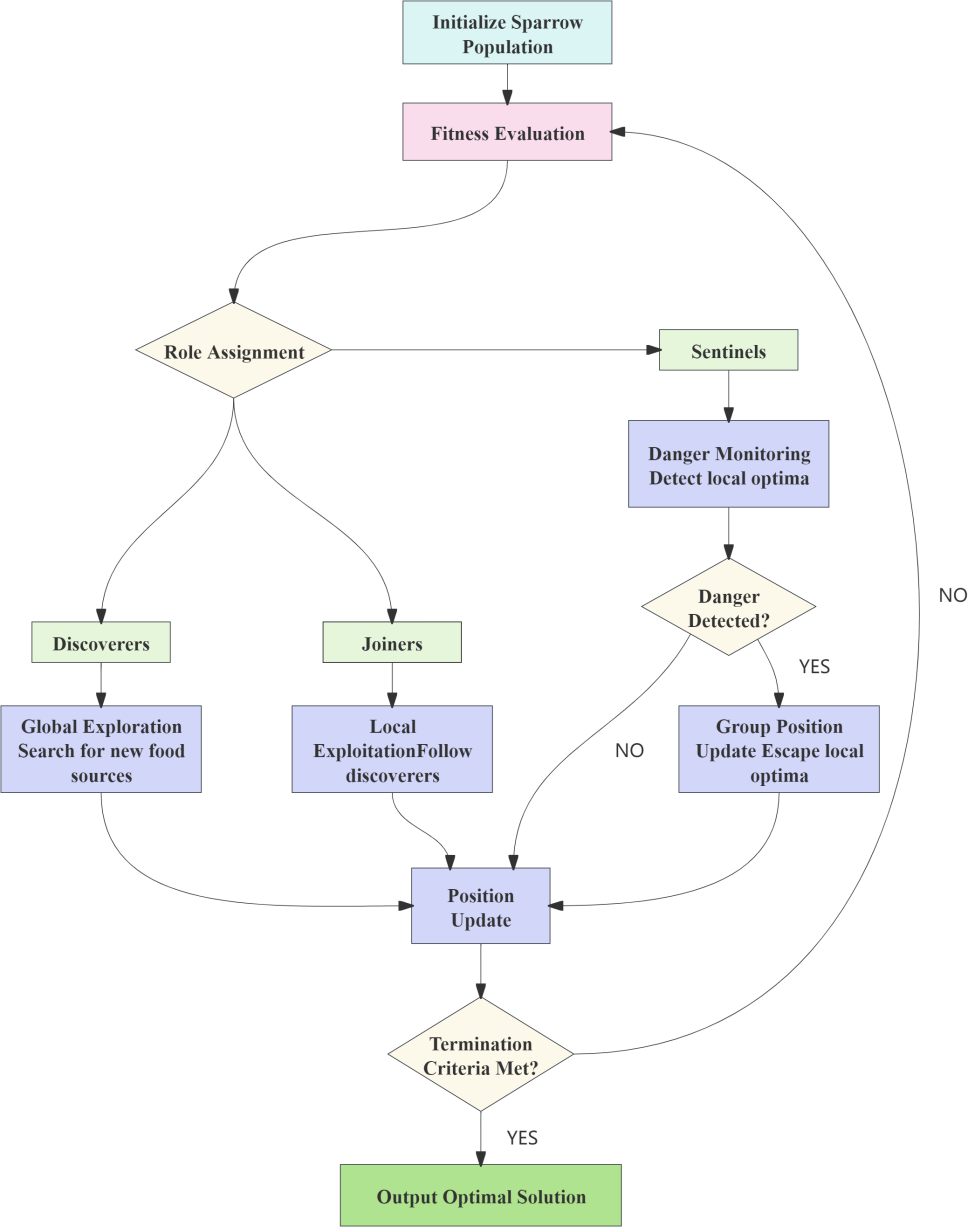
The SSA flowchart.

To overcome the inefficiency and tendency to converge to local optima of traditional hyperparameter tuning methods (e.g., grid search and random search) in high-dimensional spaces, this study employs the SSA for automated hyperparameter optimization (Xue and Shen, 2020). SSA is a metaheuristic optimization algorithm inspired by the foraging and anti-predation behaviors of sparrow groups. In this model, the sparrow population is divided into two roles: scouts, responsible for exploring new food sources (i.e., potential optimal solutions in the search space), and followers, who follow the scouts and utilize existing information. Additionally, some sparrows act as sentinels, triggering group position updates upon sensing danger (such as getting stuck in a local optimum), thereby enhancing the ability to escape local optima. By simulating this social behavior mechanism, SSA achieves a balance between global exploration and local exploitation, making it suitable for complex, non-convex, high-dimensional optimization problems, such as hyperparameter tuning in machine learning. The flowchart is shown in **Figure 2**.

SSA is a population-based metaheuristic algorithm inspired by the foraging and anti-predation behaviors of sparrows, known for its strong global search capability and rapid convergence. In this work, SSA is applied to optimize key hyperparameters of each model, with the objective of maximizing the composite evaluation metric PRFA—a weighted average of Precision, Recall, F1-score, and Accuracy (with equal weights)—on the validation set. This objective function is designed to balance classification performance across all severity levels, particularly improving detection accuracy for minority classes (e.g., mild disease cases). Although the F1-score inherently integrates Precision and Recall, the practical application context of this study—early detection of mild crop diseases—entails diverse performance priorities among different stakeholders: agronomists prioritize minimizing missed diagnoses (high Recall), system operators emphasize the reliability of alerts (high Precision), and managers require a balanced view of overall classification accuracy (Accuracy). Therefore, the PRFA metric is not intended to be a theoretically non-redundant evaluation measure; rather, it serves as a compromise proxy metric that reflects the multi-stakeholder requirements and guides the hyperparameter optimization process toward a balanced trade-off across multiple performance dimensions.

**Figure 3:**
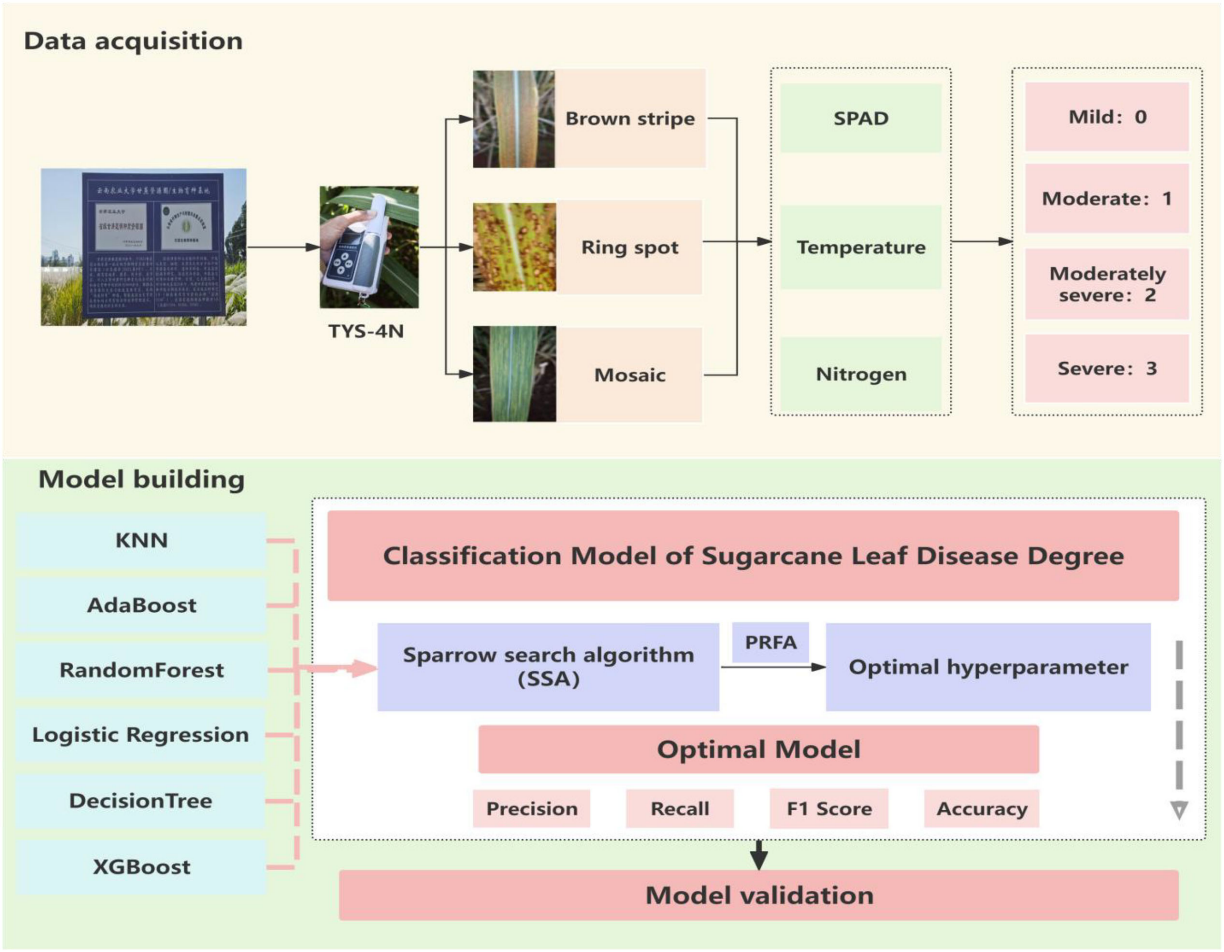
Technical workflow.

The entire optimization process is conducted within a cross-validation framework (e.g., 5-fold CV) to ensure stable and generalizable performance estimation. The final optimized models are then evaluated on an independent test set and used to construct the physiological trait-based model for sugarcane disease severity assessment. The overall technical workflow is illustrated in **Figure 3**.

All experiments were performed on a computer equipped with an AMD Ryzen 7 4800H with Radeon Graphics (2.90 GHz), using Python 3.9. The primary software libraries include Scikit-learn 1.6.0, XGBoost 2.1.3, NumPy, Pandas, and a custom-developed SSA optimization framework.

Within the SSA framework, hyperparameter optimization for each machine learning model is conducted over a predefined search space. Each individual in the sparrow population represents a candidate hyperparameter combination, and the algorithm iteratively updates their positions to maximize the PRFA score on the validation set, which serves as the fitness function. The algorithm is configured with a maximum of 100 iterations and a population size of 30. Early stopping is applied if the fitness value does not improve for 10 consecutive generations. To enhance computational efficiency, parallel execution is enabled via the n_jobs=-1 parameter in scikit-learn, leveraging all available CPU cores.

Selected hyperparameters and their corresponding search ranges are listed in **Table 2**. A fixed random seed (random_state=42) was used for reproducibility, while remaining hyperparameters were set to default values. All hyperparameters were encoded (either continuously or discretely) into the SSA search vector, with boundary constraints and type validation enforced during optimization. Ultimately, the optimal hyperparameter combination yielding the highest PRFA score is selected for each model and used in subsequent performance evaluation on the independent test set.

**Table 2:** Selected hyperparameters and their search spaces for the base model.

Model	Hyperparameter	Search space/range
KNN	n_neighbors	[1, 2, 3, 4, 5, 6, 7, 8, 9, 10]
	weights	[‘uniform’, ‘distance’]
	algorithm	[‘auto’, ‘ball_tree’, ‘kd_tree’, ‘brute’]
	p	[1, 2, 3, 4, 5]
	leaf_size	[10, 20, 30, 40, 50]
AdaBoost	n_estimators	[10, 20, 30, 40, 50, 60, 70, 80, 90, 100]
	learning_rate	[0.1, 0.5, 1.0, 1.5, 2.0]
RandomForest	n_estimators	[10, 20, 30, 40, 50, 60, 70, 80, 90, 100]
	max_depth	[None, 10, 20, 30, 40, 50, 60, 70, 80, 90, 100]
	min_samples_split	[2, 3, 4, 5, 6, 7, 8, 9, 10]
	min_samples_leaf	[1, 2, 3, 4, 5, 6, 7, 8, 9, 10]
	max_features	[None, ‘sqrt’, ‘log2’]
	criterion	[“gini”, “entropy”, “log_loss”]
LogisticRegression	C	[1, 2, 3, 4, 5, 6, 7, 8, 9, 10, 11, 12, 13, 14, 15, 16, 17, 18, 19, 20]
	max_iter	[10, 20, 30, 40, 50, 60, 70, 80, 90, 100]
	solver	[‘lbfgs’, ‘liblinear’, ‘newton-cg’, ‘newton-cholesky’, ‘sag’, ‘saga’]
DecisionTree	max_depth	[None, 10, 20, 30, 40, 50, 60, 70, 80, 90, 100]
	min_samples_split	[2, 3, 4, 5, 6, 7, 8, 9, 10]
	min_samples_leaf	[1, 2, 3, 4, 5, 6, 7, 8, 9, 10]
	max_features	[None, ‘sqrt’, ‘log2’]
	criterion	[“gini”, “entropy”, “log_loss”]
	splitter	[‘best’, ‘random’]
XGBoost	n_estimators	[10, 20, 30, 40, 50, 60, 70, 80, 90, 100]
	learning_rate	[0.1, 0.5, 1.0, 1.5, 2.0]
	max_depth	[None, 10, 20, 30, 40, 50, 60, 70, 80, 90, 100]
	subsample	[0.1, 0.2, 0.3, 0.4, 0.5, 0.6, 0.7, 0.8, 0.9, 1.0]
	colsample_bytree	[0.1, 0.2, 0.3, 0.4, 0.5, 0.6, 0.7, 0.8, 0.9, 1.0]
	min_child_weight	[1, 3, 5, 7, 9, 11]
	gamma	[0, 0.1, 0.2, 0.3, 0.4, 0.5, 0.6, 0.7, 0.8, 0.9, 1.0]
	reg_alpha	[0, 0.1, 0.2, 0.3, 0.4, 0.5, 0.6, 0.7, 0.8, 0.9, 1.0]
	reg_lambda	[0, 0.1, 0.2, 0.3, 0.4, 0.5, 0.6, 0.7, 0.8, 0.9, 1.0]

### 2.3 Model evaluation metrics

To comprehensively evaluate the performance of different machine learning models in the sugarcane disease severity classification task, this study employs multiple classification evaluation metrics, including Accuracy, Precision, Recall, F1-score, and a custom composite metric named PRFA. All metrics are computed based on the confusion matrix constructed from the predicted labels and true labels on the test set.

#### 2.3.1 Accuracy

Accuracy represents the proportion of correctly classified samples among the total number of samples. It is a widely used overall performance metric suitable for most classification tasks. The formula for accuracy is defined as:


$$\text{Accuracy}=\frac{\text{TP}+\text{TN}}{\text{TP}+\text{TN}+\text{FP}+\text{FN}}$$

#### 2.3.2 Precision

Precision is the ratio of true positive predictions to all samples predicted as positive. It reflects the model’s ability to avoid false alarms when identifying diseased samples. The precision for each class is calculated as:


$$\text{Precision}=\frac{\text{TP}}{\text{TP}+\text{FP}}$$

#### 2.3.3 Recall

Recall, also known as True Positive Rate (TPR) or sensitivity, measures the proportion of actual positive samples that are correctly identified by the model. It indicates the model’s capacity to detect all instances of a given severity level. Recall is computed as:


$$\text{Recall}=\frac{\text{TP}}{\text{TP}+\text{FN}}$$

#### 2.3.4 F1-score

The F1-score is the harmonic mean of Precision and Recall, providing a balanced assessment of model performance, especially in the presence of class imbalance. The F1-score ranges from 0 to 1, with values closer to 1 indicating better performance. It is calculated as:


$$\text{F}1\text{Score}=2\cdot \frac{\text{Precision}\cdot \text{Recall}}{\text{Precision}+\text{Recall}}$$

#### 2.3.5 Composite performance metric

To balance the trade-offs among Precision, Recall, F1-score, and Accuracy, this study proposes a custom composite metric, PRFA, which computes the equally weighted average of these four metrics:


$$\text{PRFA}=\frac{1}{4}\left(\text{Precision}+\text{Recall}+\text{F}1\text{ Score}+\text{Accuracy}\right)$$

This metric ensures a holistic evaluation of model performance across multiple dimensions, particularly enhancing sensitivity to minority classes while maintaining overall classification consistency.

Definitions of Confusion Matrix Components:

TP (True Positive): Number of samples that are actually positive and correctly predicted as positive.
TN (True Negative): Number of samples that are actually negative and correctly predicted as negative.
FP (False Positive): Number of samples that are actually negative but incorrectly predicted as positive.
FN (False Negative): Number of samples that are actually positive but incorrectly predicted as negative.

## 3 Results

### 3.1 Distribution analysis of sugarcane leaf disease severity

**Figure 4:**
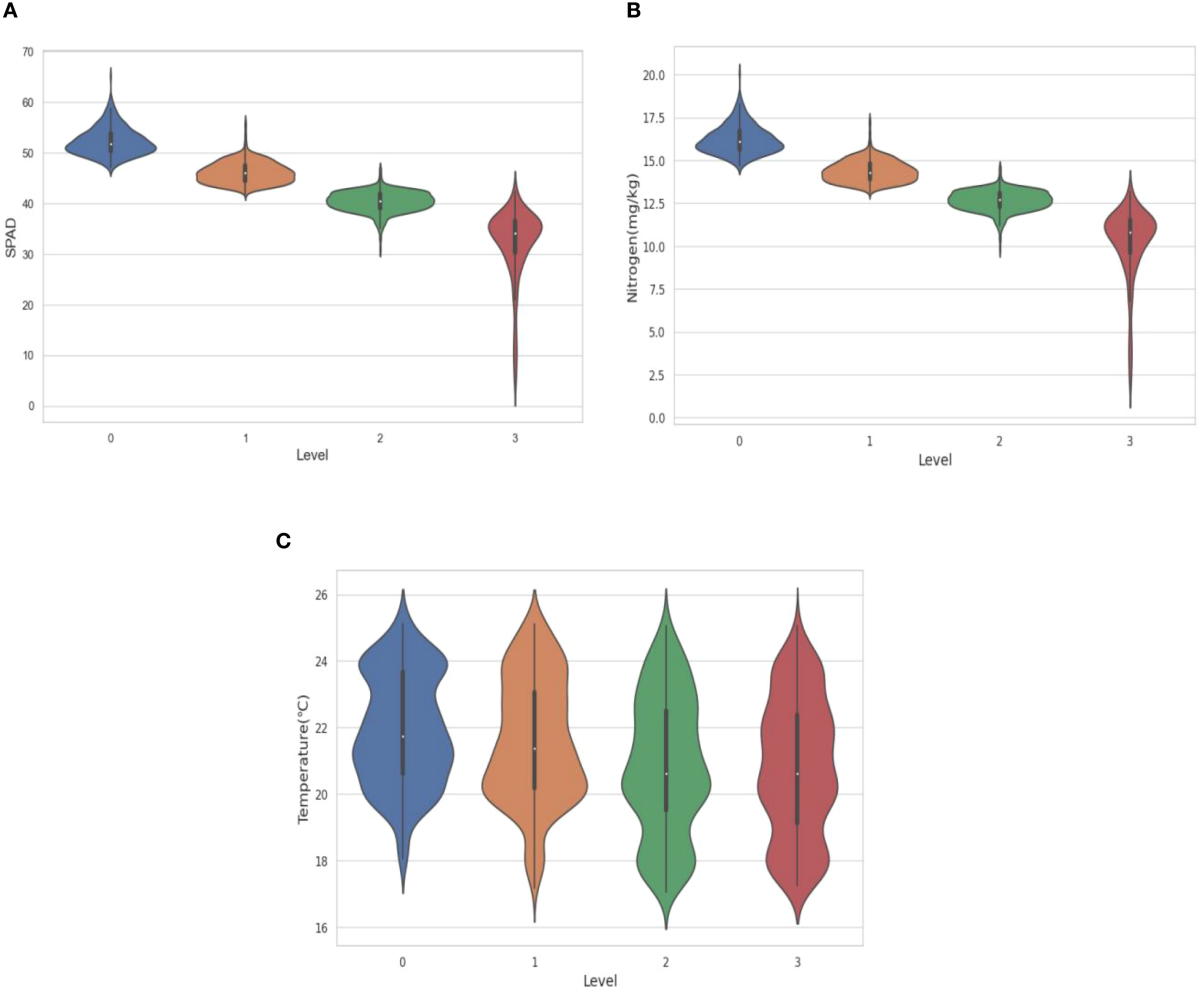
Distribution of SPAD values, leaf surface temperature, and nitrogen content across different sugarcane disease severity levels (Level 0–3). **(A)** shows the distribution of SPAD values; **(B)** shows the distribution of nitrogen content; **(C)** shows the distribution of leaf surface temperature.

**Figure 4** illustrates the distribution patterns of SPAD values, leaf surface temperature, and nitrogen content in sugarcane leaves across disease severity levels (Level 0–3), visualized using violin plots. These plots effectively capture the central tendency, dispersion, and skewness of each variable within severity classes, providing insights into their response to disease progression.

#### 3.1.1 SPAD value distribution

**Figure 3A** and **Table 3** illustrate the distribution patterns of SPAD values across different disease severity levels. At Level 0, SPAD values are primarily distributed between 47 and 58, with a median of approximately 51, indicating that chlorophyll content in healthy or slightly affected leaves is concentrated at higher levels. As the disease severity increases, SPAD values show a systematic downward trend, with the distribution becoming wider but shorter. By Level 3, the range of SPAD values decreases to its lowest point, with the height of the distribution significantly increasing but the width becoming narrower, indicating that the data is highly concentrated around the median but spans a larger overall range, with some low-value outliers. This reflects the individual variability in chlorophyll degradation under severe disease conditions.

**Table 3:** Distribution ranges and medians of SPAD values across different disease severity levels.

Disease severity levels	Distribution ranges	Medians
Level 0 (mild)	47–58	51
Level 1 (moderate)	42–52	45
Level 2 (moderately severe)	35–45	40
Level 3 (severe)	21–42	35

Overall, both the median and interquartile range of SPAD values decrease monotonically with disease progression, demonstrating a strong negative correlation between chlorophyll content and disease severity. Except for Level 3, the distributions in the first three levels are relatively symmetric and compact, suggesting stable physiological responses in early to mid-stage infections.

#### 3.1.2 Nitrogen content distribution

**Figure 3B** and **Table 4** illustrate the evolution patterns of nitrogen content as the disease progresses. At Level 0, nitrogen content is concentrated between 15 and 18 mg/kg, with a median of approximately 16 mg/kg, showing a symmetric and dense distribution. Similar to the trend observed for SPAD values, as disease severity increases, the range, median, and height of the nitrogen content distribution all gradually decrease, while the width of the distribution becomes wider. By Level 3, both the range and median of the distribution have reached their lowest points, with the distribution becoming narrower but showing significant tailing, particularly at the lower end (<10 mg/kg), where there is a notable extension of density. This indicates that severe disease leads to substantial nitrogen depletion and an increased variability among individuals.

**Table 4:** Distribution ranges and medians of nitrogen content across different disease severity levels.

Disease severity levels	Distribution ranges (mg/kg)	Medians(mg/kg)
Level 0 (mild)	15–18	16
Level 1 (moderate)	13–16	14.5
Level 2 (moderately severe)	11–14	12.5
Level 3 (severe)	7–13	10.5

These results confirm a continuous decline in nitrogen content with disease progression, with distribution morphology transitioning from symmetric and concentrated to skewed and dispersed in advanced stages. This supports nitrogen content as a sensitive indicator of disease severity.

#### 3.1.3 Leaf surface temperature distribution

**Figure 4C** displays the leaf temperature distribution across severity levels. In Level 0, temperatures range from 18°C to 25°C, with a median of 20–22°C. From Level 1 to Level 3, the overall range remains largely unchanged (17–25°C), and the median shows only a slight downward trend, indicating a weak response of leaf temperature to disease progression.

Notably, all severity levels exhibit multi-modal and asymmetric distributions, with multiple density peaks and unequal tails. This suggests substantial intra-class variability, likely influenced by non-disease factors such as microclimate, stomatal conductance, or water stress. Consequently, leaf temperature alone demonstrates limited discriminative power compared to SPAD and nitrogen content, highlighting its limited utility as a standalone diagnostic feature.

### 3.2 Training and optimization of classification models

Six machine learning models—KNN, AdaBoost, RF, LR, DT, and XGBoost—were trained to classify sugarcane disease severity based on SPAD, temperature, and nitrogen data collected from plants infected with brown stripe, ring spot, and mosaic diseases. Hyperparameters were optimized using the SSA to enhance model performance and generalization. The dataset was split into training (90%) and testing (10%) sets to ensure sufficient training and independent evaluation.

With default hyperparameters, the models were evaluated on the test set using Precision, Recall, F1-score, Accuracy, and PRFA (see **Table 5**). Logistic Regression (LR) achieved the best performance among default models, with Precision=0.9154, Recall=0.9144, F1-score=0.9145, and Accuracy=0.9144, indicating high consistency and stability. In contrast, AdaBoost performed the worst (Precision=0.5898, Recall=0.6216, F1-score=0.5367), reflecting its sensitivity to class imbalance and suboptimal default settings. KNN achieved scores close to LR (>0.90), while RF and XGBoost showed robust performance (~0.89). DT scored ~0.85—29% higher than AdaBoost—demonstrating acceptable baseline performance.

**Table 5:** Comparison of model performance before and after optimization.

Operation	Model	Precision	Recall	F1 Score	Accuracy
Unoptimized	KNN	0.9052	0.9054	0.9051	0.9054
	AdaBoost	0.5898	0.6216	0.5367	0.6216
	RF	0.8964	0.8964	0.8961	0.8964
	LR	0.9154	0.9144	0.9145	0.9144
	DT	0.8551	0.8559	0.8551	0.8559
	XGBoost	0.8918	0.8919	0.8917	0.8919
Optimized	KNN	0.8965	0.8964	0.8959	0.8964
	AdaBoost	0.7589	0.7162	0.6804	0.7162
	RF	0.9161	0.9144	0.9141	0.9144
	LR	0.9163	0.9144	0.9145	0.9144
	DT	0.8940	0.8919	0.8912	0.8919
	XGBoost	0.9199	0.9189	0.9186	0.9189

SSA significantly improved the performance of all models by optimizing key hyperparameters to maximize PRFA on a validation subset. After optimization: XGBoost emerged as the top performer, achieving Precision=0.9199, Recall=0.9189, F1-score=0.9186, Accuracy=0.9189, surpassing even the unoptimized LR model. Improvements ranged from +0.027 to +0.028 across metrics, demonstrating SSA’s effectiveness in fine-tuning ensemble models. AdaBoost showed the most dramatic improvement: F1-score increased by 0.1043, with all metrics converging toward 0.67–0.69, indicating enhanced stability and reduced bias due to better parameter configuration. KNN, RF, LR, and DT also improved by 0.01–0.03 on average, confirming the broad applicability of SSA in enhancing model robustness.

To clearly illustrate the relative effectiveness of different optimization strategies, this study directly compares the original XGBoost model (accuracy: 89.19%, F1-score: 89.17%) with various models optimized by the Sparrow Search Algorithm (SSA). The results show that SSA-XGBoost achieves the best performance among all compared models, with an accuracy improvement of 2.70 percentage points over the baseline XGBoost. Among the other SSA-optimized models, SSA-LR and SSA-RF both attain an accuracy of 91.44% (F1-scores of 91.45% and 91.41%, respectively), slightly lower than SSA-XGBoost. SSA-DT achieves an accuracy of 89.19%, comparable to the baseline XGBoost, while SSA-KNN (89.64%) and SSA-AdaBoost (71.62%) show no clear advantage. In summary, XGBoost optimized by SSA not only significantly outperforms its original version but also maintains a leading position in comparison with other SSA-optimized models, demonstrating superior overall classification capability.

The PRFA metric, defined as the equally weighted average of the four core metrics, was used to rank model performance. As summarized in **Table 6**, the SSA-XGBoost model achieved the highest PRFA of 0.9326, outperforming all other models. This indicates superior overall performance in balancing precision, recall, and accuracy across severity levels.

**Table 6:** PRFA scores and optimal hyperparameters of the six machine learning models after SSA optimization.

Model	PRFA	Hyperparameter
KNN	0.9189	{‘n_neighbors’: 5, ‘weights’: ‘distance’, ‘algorithm’: ‘ball_tree’, ‘p’: 2, ‘leaf_size’: 50}
AdaBoost	0.9016	{‘n_estimators’: 80, ‘learning_rate’: 1.0}
RF	0.9191	{‘n_estimators’: 80, ‘max_depth’: 50, ‘min_samples_split’: 6, ‘min_samples_leaf’: 9, ‘max_features’: ‘sqrt’, ‘criterion’: ‘log_loss’}
LR	0.9149	{‘C’: 11, ‘max_iter’: 140, ‘solver’: ‘liblinear’}
DT	0.9236	{‘max_depth’: 70, ‘min_samples_split’: 5, ‘min_samples_leaf’: 8, ‘max_features’: ‘log2’, ‘criterion’: ‘gini’, ‘splitter’: ‘random’}
XGBoost	0.9326	{‘n_estimators’: 90, ‘learning_rate’: 0.1, ‘max_depth’: 70, ‘subsample’: 0.2, ‘colsample_bytree’: 0.4, ‘min_child_weight’: 5, ‘gamma’: 1.0, ‘reg_alpha’: 0.9, ‘reg_lambda’: 0.8}

**Figure 5:**
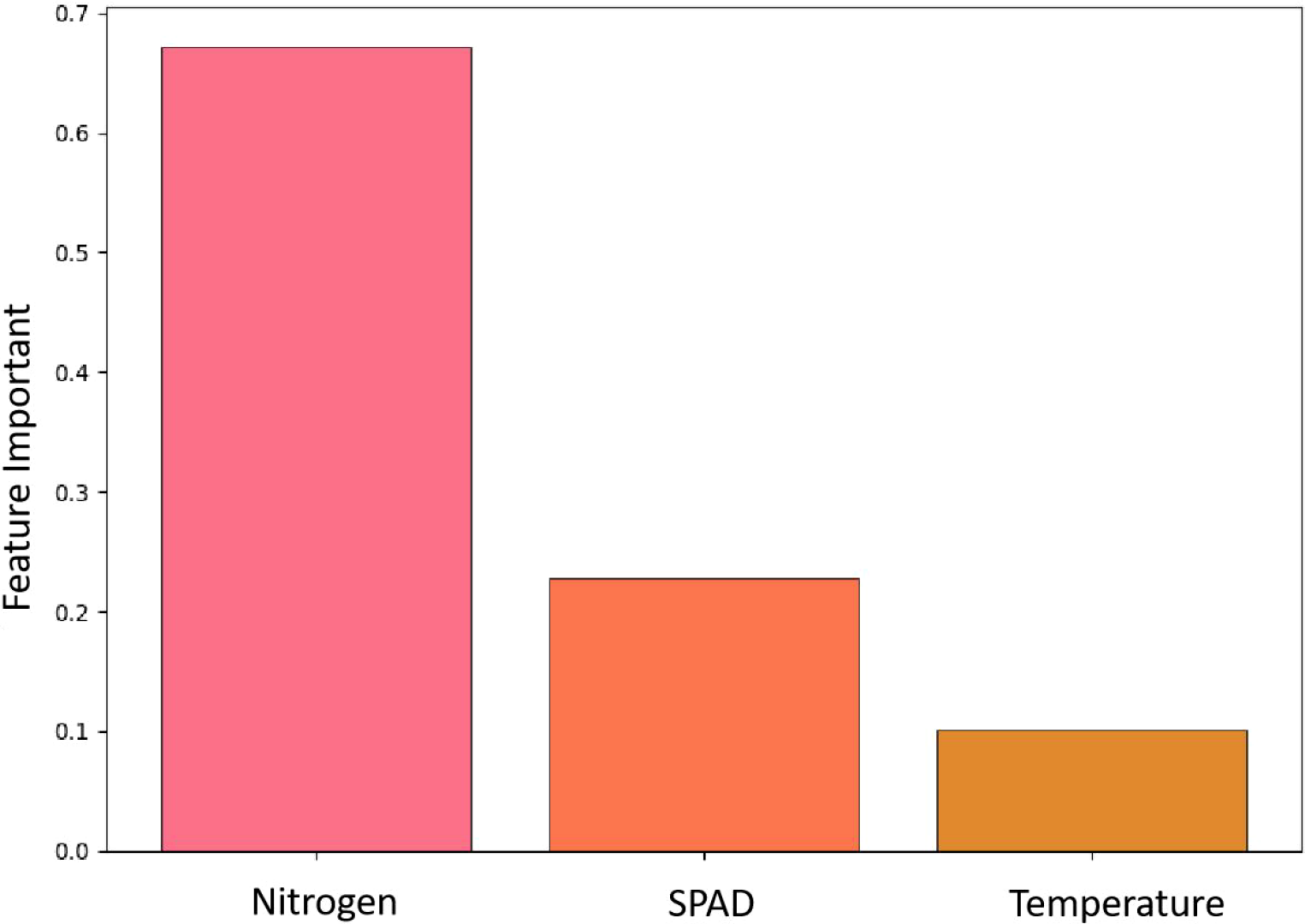
The feature importance results of the SSA-XGBoost model.

In this study, we evaluated the contribution of each feature to the predictive performance of the SSA-XGBoost model by computing feature importance scores using the model’s `feature_importances_` attribute and visualized the top 20 features in a bar plot (see **Figure 5**). As shown in the figure, among all physiological features related to sugarcane disease, nitrogen content exhibited a significantly higher importance score than both SPAD and leaf temperature, while leaf temperature ranked lowest and contributed minimally. This finding is highly consistent with our qualitative observations from the disease severity distribution analysis—namely, that SPAD and nitrogen concentration effectively capture the gradient of disease severity, whereas leaf temperature shows limited discriminative power across severity levels.

### 3.3 External validation and generalization analysis

To evaluate real-world applicability, the optimized SSA-XGBoost model was externally validated on an independent dataset of 635 samples collected from the Gengma Sugarcane Plantation. The dataset includes 28 (Level 0), 63 (Level 1), 127 (Level 2), and 417 (Level 3) samples, reflecting the field-realistic increasing prevalence of severe disease.

As shown in **Table 7**, the model achieved an overall Accuracy of 0.91, with a weighted F1-score of 0.91 and a macro F1-score of 0.89, indicating strong generalization. Specifically: Precision was highest for Level 2 (0.92) and lowest for Level 0 (0.88), suggesting high specificity for moderately severe cases. Recall was perfect for Level 0 (1.00), indicating no missed detection of healthy/lightly diseased plants, but lowest for Level 2 (0.65), revealing misclassification or under-detection. F1-score was highest for Level 3 (0.95) and lowest for Level 2 (0.76), highlighting classification ambiguity in the moderately severe category.

**Table 7:** Classification report on the validation dataset.

Class	Precision	Recall	F1-score	Support
0	0.88	1.00	0.93	28
1	0.91	0.92	0.91	63
2	0.92	0.65	0.76	127
3	0.91	0.99	0.95	417
accuracy	–	–	0.91	635
macro avg	0.90	0.89	0.89	635
weighted avg	0.91	0.91	0.91	635

**Figure 6:**
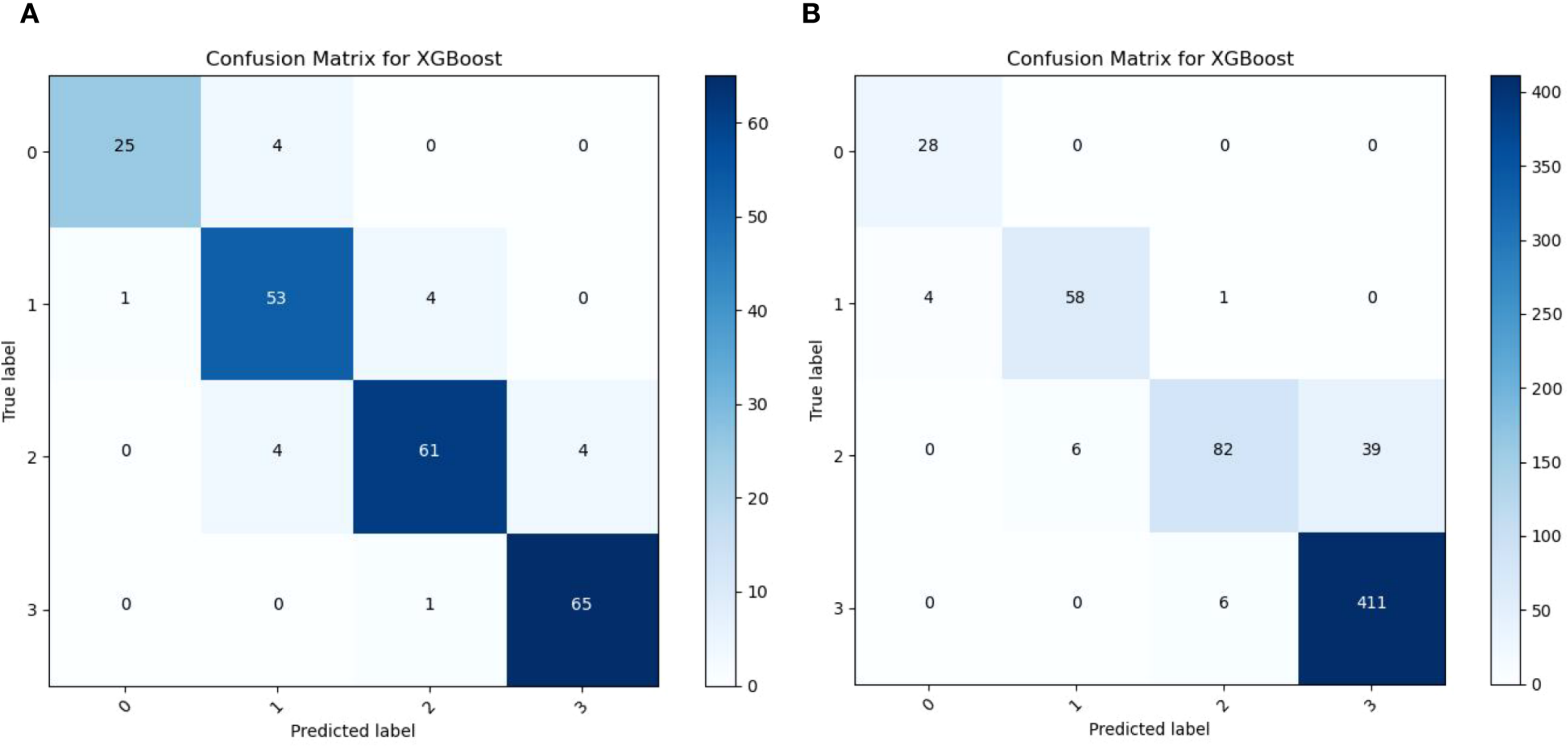
Confusion matrices on the original test set and the independent validation set. **(A)** is the confusion matrix of the optimized XGBoost in the original test set; **(B)** is the confusion matrix of the optimized XGBoost in the independent validation set.

To further assess performance stability, confusion matrices were generated for both the original test set and the independent validation set (see **Figure 6**). Both datasets yielded an overall accuracy of 0.91, confirming model consistency. On the original test set, classification was balanced: Level 0 accuracy=86.21%, Level 1 = 93.02%, Level 2 F1 = 88.41%, Level 3 = 98.48%. On the Gengma validation set, Level 0 recall reached 100%, and Level 3 precision was 98.56%, confirming robust detection of healthy and severely diseased plants.

However, Level 2 recall dropped to 64.57% (F1 = 76.34%), with many samples misclassified as Level 3. This suggests symptom overlap and transitional characteristics between moderately severe and severe disease stages in real-field conditions.

This performance gap indicates that while the current physiological features (SPAD, temperature, nitrogen) are effective for detecting extreme disease states, they struggle to distinguish transitional stages (Level 2), likely due to overlapping symptom expression and environmental noise.

The SSA-XGBoost model demonstrates superior performance in classifying sugarcane disease severity using physiological traits, achieving high accuracy and strong generalization, particularly for healthy (Level 0) and severely infected (Level 3) plants. SPAD values and nitrogen content are identified as highly sensitive and reliable indicators of disease progression, while leaf temperature exhibits limited discriminative power due to high intra-class variability. External validation confirms the model’s robustness in real-world conditions. However, classification of moderately severe cases (Level 2) remains challenging due to ambiguous symptom expression, suggesting the need for increased sampling during transitional stages, integration of temporal or multi-modal data (e.g., canopy imaging, weather variables), and advanced feature engineering to improve boundary discrimination. Overall, this study validates the feasibility of deploying SSA-optimized XGBoost models for large-scale, non-destructive monitoring of sugarcane health across diverse agro-ecological environments.

## 4 Discussion

The current study introduces a novel approach for classifying the severity of sugarcane leaf diseases by integrating physiological features with machine learning techniques, aiming to address challenges in early detection and classification accuracy within disease management. By leveraging key indicators such as SPAD values, leaf surface temperature, and nitrogen content acquired through portable plant nutrient analyzers (TYS-4N), six classification models—KNN, AdaBoost, RF, LR, DT, and XGBoost—were constructed. The SSA was employed for hyperparameter optimization, significantly enhancing model performance.

### 4.1 Biological basis of selected features

The chosen physiological traits are grounded in biological principles. SPAD values, reflecting chlorophyll content, typically decrease due to damage to chloroplast structure during disease progression. Leaf surface temperatures are influenced by stomatal conductance and transpiration rates, often rising as stomata close upon infection, reducing heat dissipation. Nitrogen levels directly affect plant growth and disease resistance, showing systematic changes throughout disease development. These metrics provide stable and sensitive inputs for classification models, contrasting with deep learning approaches that rely on image data. Numerical physiological parameters obtained via portable devices offer advantages like ease of collection, robustness against environmental interference, and minimal preprocessing requirements, making them more suitable for rapid field detection and practical application.

Nevertheless, this study has two key limitations that should be acknowledged. First, the current models focus exclusively on disease severity grading and do not distinguish among specific sugarcane disease types (e.g., brown stripe, ring spot, or mosaic). Future work should incorporate visual, spectral, or molecular signatures to enable precise pathogen identification alongside severity assessment.

Second—and equally important—the set of physiological features is limited to only three parameters: SPAD value, leaf surface temperature, and nitrogen content. While these are biologically meaningful and field-accessible, they capture only a partial view of the plant’s stress response. Additional physiological indicators, such as leaf water potential, relative chlorophyll fluorescence (e.g., Fv/Fm), or leaf moisture content, reflect complementary mechanisms of plant defense and could significantly enhance model sensitivity—particularly to early-stage infections that may not yet manifest in SPAD or nitrogen changes.

Looking forward, integrating these physiological traits with multimodal data sources—such as hyperspectral imaging, thermal infrared sensing, or even metabolomic profiles—could unlock a more holistic understanding of plant health. Such a multi-layered approach would not only improve predictive accuracy but also support earlier and more robust diagnosis under diverse field conditions, paving the way for next-generation precision disease management systems in sugarcane and other crops.

### 4.2 Enhanced model performance through SSA optimization

The SSA algorithm’s global search capabilities and fast convergence properties were leveraged to optimize critical hyperparameters across all six machine learning models. This approach effectively mitigates the risk of local optima inherent in conventional tuning methods such as grid or random search. Experimental results demonstrated significant improvements in classification accuracy and stability after optimization, with SSA-XGBoost achieving the best overall performance (Precision, Recall, F1 Score, and Accuracy all exceeding 0.9186; PRFA=0.9326), thereby confirming SSA’s effectiveness in enhancing model performance.

Notably, XGBoost exhibited greater performance gains from SSA optimization compared to Random Forest (RF), Logistic Regression (LR), and Decision Tree (DT). This can be attributed to XGBoost’s algorithmic structure: as a gradient boosting framework, it relies on a complex set of interdependent hyperparameters—such as learning rate, maximum tree depth, subsample ratio, and L1/L2 regularization—that jointly control model complexity, bias-variance trade-off, and generalization. The global exploration ability of SSA is particularly well-suited to navigating this high-dimensional, non-convex hyperparameter space, enabling XGBoost to fully exploit its capacity for modeling nonlinear relationships and high-order feature interactions—common in agricultural physiological data (e.g., SPAD, leaf temperature, nitrogen content).

In contrast, LR has limited expressiveness due to its linear nature and few tunable parameters; DT, while interpretable, lacks robustness and is prone to overfitting without ensemble strategies; and RF, though inherently stable through bagging and random feature selection, exhibits reduced sensitivity to hyperparameter tuning because of its stochastic design. Consequently, the synergy between SSA’s efficient global search and XGBoost’s flexible, high-capacity architecture yields more substantial performance improvements than with other base learners, highlighting the importance of aligning optimization strategies with model-specific characteristics.

### 4.3 Advantages over deep learning methods

Compared to deep learning alternatives, our method exhibits several practical advantages. Firstly, it requires minimal annotated image data, relying instead on a small set of biologically interpretable physiological parameters (e.g., SPAD, leaf temperature, nitrogen content) to achieve high-precision severity grading—thereby significantly reducing data acquisition costs and eliminating the need for labor-intensive pixel-level or image-level labeling. Secondly, the lightweight model architecture and fast training/inference speeds make it well-suited for deployment in resource-constrained agricultural environments, such as on edge devices in sugarcane fields. Moreover, the transparency of input features and model decisions enhances interpretability, fostering greater trust among agronomists and end-users.

That said, recent comparative studies (e.g., Maurya et al., 2023; Gupta et al., 2024) highlight that deep learning models—particularly convolutional neural networks (CNNs) and vision transformers—excel at discriminating between visually distinct disease types from leaf images, a capability our current physiology-only framework does not address. To bridge this gap, we envision a hybrid diagnostic system that synergistically combines the strengths of both paradigms: a deep learning module could first identify the specific disease type (e.g., brown stripe *vs*. mosaic) from RGB or hyperspectral images, while a physiology-driven module (such as SSA-XGBoost) would then assess the severity level based on real-time field measurements of SPAD, temperature, and nitrogen.

Such a two-stage or multi-branch architecture would enable comprehensive disease diagnosis—simultaneously answering “what disease is present?” and “how severe is it?”—while leveraging the robustness of image-based recognition and the field-deployability of physiological sensing. This integrated approach represents a promising direction for future work, aligning with emerging trends in multimodal plant health monitoring.

### 4.4 Challenges in classifying moderately severe disease (class 2)

Class 2 (moderately severe) samples achieved a recall of only 64.57% and an F1-score of 76.34%, significantly lower than other severity levels, with the majority misclassified as Class 3. This performance gap suggests that the model struggles to reliably identify cases at the intermediate stage of disease progression, a critical window for timely intervention.

This limitation stems not merely from visual or symptomatic similarity between Class 2 and Class 3 under field conditions, but more fundamentally from the insufficient representational capacity of the current physiological feature set during the transitional phase of disease development. While SPAD, leaf temperature, and nitrogen content effectively differentiate healthy (Class 0) and severely diseased (Class 3) plants, their response patterns tend to plateau or change nonlinearly as symptoms advance from moderate to severe, resulting in ambiguous decision boundaries in the feature space. The problem is further highlighted in cross-dataset validation: Class 2 performance remains relatively stable on the internal test set but declines markedly in the external validation set collected under real-world, complex field conditions, indicating limited robustness to environmental perturbations—such as variations in light and humidity—and natural inter-plant heterogeneity.

To address these challenges, future efforts could focus on enriching the input representation through multiple complementary strategies. Incorporating dynamic physiological indicators—such as daily SPAD decline rates or diurnal leaf temperature ranges—along with higher-order feature interactions (e.g., SPAD × nitrogen content) may better capture the temporal dynamics of disease progression. Simultaneously, increasing sampling density during the peak occurrence of Class 2 symptoms or in representative field plots, combined with resampling or cost-sensitive learning techniques, could help mitigate class boundary ambiguity. Most promisingly, fusing canopy-scale imaging data from UAV-based RGB or multispectral sensors—providing texture, color, and structural phenotypic cues—with micro-meteorological variables such as temperature, humidity, and precipitation would enable a more holistic “physiology–phenotype–environment” modeling framework.

Although the current model demonstrates strong performance for healthy and severely diseased cases—making it well-suited for large-scale screening and early warning—its accuracy for moderately severe disease remains a key bottleneck. By implementing these integrated approaches, future systems could evolve from coarse-grained severity grading toward fine-grained, context-aware diagnosis, ultimately supporting more precise and actionable crop protection strategies in real-world agricultural environments.

## 5 Conclusion

This study proposes an intelligent classification methodology for determining the severity of sugarcane leaf diseases using physiological characteristics combined with machine learning. Key physiological indicators including SPAD values, leaf surface temperature, and nitrogen content were collected to construct six classification models: KNN, AdaBoost, RF, LR, DT, and XGBoost. Hyperparameter optimization was conducted using the SSA. Results indicate that the SSA-XGBoost model outperformed others on the test set, with evaluation metrics exceeding 0.9186 and a PRFA score of 0.9326. In the independent validation set from Gengma County, the overall accuracy reached 0.91, demonstrating excellent generalization ability and field applicability.

Compared to deep learning models, our approach offers distinct advantages in terms of data acquisition convenience, computational efficiency, and model interpretability, making it highly suitable for rapid diagnostics and early warning in agricultural settings. This study provides an effective technological pathway for intelligent management of sugarcane diseases and offers a replicable methodology for precise identification of other crop diseases.

## Data Availability

The original contributions presented in the study are included in the article/**Supplementary Material**. Further inquiries can be directed to the corresponding authors.
