# Correction: Intelligent grading of sugarcane leaf disease severity by integrating physiological traits with the SSA-XGBoost algorithm

**DOI:** 10.3389/fpls.2025.1727382

**Published:** 2025-11-18

**Authors:** Xinrui Wang, Jihong Sun, Peng Tian, Mengyao Wu, Jiawei Zhao, Jiangquan Chen, Ye Qian, Canyu Wang

**Affiliations:** 1 College of Big Data, Yunnan Agricultural University, Kunming, Yunnan, China; 2 College of Information Engineering, Kunming University, Kunming, Yunnan, China; 3 Qujing Tobacco Company, Qujing, Yunnan, China

**Keywords:** sugarcane leaf diseases, disease severity grading, physiological traits, machine learning classification, hyperparameter optimization

Affiliation 3 “Qujing Tobacco Company, Qujing, Yunnan, China” was omitted for author “Mengyao Wu”. This affiliation has now been added for author “Mengyao Wu”. The **Conflict of Interest** statement has been updated to include this commercial affiliation.

The original Funding statement “The author(s) declare financial support was received for the research and/or publication of this article. This study was supported by the Yunnan Provincial Science and Technology Talent and Platform Program (Academician Expert Workstation) (202405AF140077, 202505AF350026), Yunnan Province Basic Research Special Project (202401AT070253), Yunnan Province Young and Middle aged Academic and Technical Leaders Reserve Talent Project (202405AC350108).” has been updated to “The author(s) declare financial support was received for the research and/or publication of this article. This study was supported by the Yunnan Provincial Science and Technology Talent and Platform Program (Academician) Expert Workstation) (202505AF350026, 202405AF140013).”

The original version of this article has been updated.

