# Supplementary material for: Intelligent grading of sugarcane leaf disease severity by integrating physiological traits with the SSA-XGBoost algorithm
Source: Front Plant Sci. 2025 Oct 15;16:1698808. doi: 10.3389/fpls.2025.1698808 (PMC12568666; doi:10.3389/fpls.2025.1698808)
Supplement: Supplementary file 2 [file Table2.docx]

Supplementary Material

# Supplementary Tables

**Table 1.** Grading criteria for sugarcane leaf disease severity.

| **Disease** | **Severity levels** | **Grading criteria** |
| --- | --- | --- |
| Brown Stripe | mild | Small, translucent, water-soaked spots, approximately 0.5 mm in diameter, appear on only a few young leaves. These lesions initially present as pale yellow to brownish small spots or short streaks, less than 1 cm in length and about 1 mm in width, confined to the basal region of individual leaves. |
|  | moderate | Lesions begin to extend along the veins, forming irregular brownish-yellow streaks that are 2–5 cm in length and 1–2 mm in width. The lesions have well-defined margins with a slight yellow halo surrounding them. As they progressively expand toward the mid-portion of the leaf, they do not yet cover the entire leaf surface. |
|  | moderately severe | Numerous leaves exhibit prominent brownish-yellow streak-like lesions, with some lesions turning reddish-brown. Lesions are typically longer than 5 cm and 2–4 mm in width, widely distributed across the mid- to apical regions of the leaves, affecting an increasing number of leaf blades. |
|  | severe | Leaves are densely covered with lesions that extend from the base to the apex. Lesions are typically longer than 10 cm and coalesce extensively, leading to premature leaf senescence. In some cases, apical rot is observed, indicating severe systemic infection. |
| Ring Spot | mild | Oval to elongated lesions begin to appear on a few older leaves, with no more than five per leaf. Lesions are dark green to brown, approximately 1 cm in diameter, and feature a narrow pale yellow margin. The center of each lesion is darker in color, and the overall shape is relatively regular, occurring primarily in the mid- to basal regions of the leaf. |
|  | moderate | Lesions gradually enlarge and become irregular in shape, with the number per leaf increasing to 10–20 and diameters reaching approximately 2 cm. The lesion color darkens, and occasional coalescence occurs. Lesions are primarily concentrated in the mid- to basal regions of the leaf. |
|  | moderately severe | Lesions on the leaves increase significantly in number, ranging from 30 to 50 per leaf. Their color changes from grayish-white to brown or black, with some lesions developing a yellowish center and a distinct pale red margin. |
|  | severe | Leaves are almost entirely covered with lesions, extending from the base to the apex, with more than 50 lesions per leaf. Lesions are dark brown to black in color and coalesce extensively, forming large necrotic areas. |
| Mosaic | mild | A few fine yellow streaks or spots appear at the base of new leaves, approximately 1 mm in width, typically located in the mid- to basal regions of the leaf. The chlorotic areas show only slight color contrast. |
|  | moderate | Irregular chlorotic mottling or streaks with alternating yellow and green patterns become more numerous, with 10 to 20 such streaks or patches per leaf, approximately 1–2 mm in width. These symptoms begin to extend across the entire leaf blade but remain concentrated in the basal and mid-regions. The chlorotic areas become more pronounced, resulting in a distinct yellow-green contrast. |
|  | moderately severe | Most leaves exhibit typical mosaic symptoms, with 30 to 50 streaks per leaf. The streaks or spots are distributed nearly across the entire leaf surface, extending beyond the basal and mid-regions to reach the leaf apex. |
|  | severe | Leaves are almost completely covered by dense, interlacing yellow-green streaks, with more than 50 streaks per leaf. Lesions extend from the base to the apex, and the foliage displays pronounced mosaic symptoms, with highly visible alternating yellow and green patterns. |
